# Are remittances a buffer against food insecurity? Lessons from a national survey in Bangladesh

**DOI:** 10.1371/journal.pone.0334391

**Published:** 2025-10-17

**Authors:** Md. Muhitul Alam, Faria Rauf Ria, Mohaimen Mansur, Md. Azad Uddin, Md. Israt Rayhan

**Affiliations:** 1 Institute of Statistical Research and Training, University of Dhaka, Dhaka, Bangladesh; 2 Department of Mathematics and Natural Sciences, BRAC University, Dhaka, Bangladesh; 3 Additional Director, Bangladesh Bank, Bangladesh; Southern Illinois University Carbondale, UNITED STATES OF AMERICA

## Abstract

Food insecurity continues to be a major global challenge, affecting many people worldwide. Bangladesh is particularly vulnerable due to its susceptibility to frequent climate shocks and socioeconomic challenges. This study investigates the causal relationship between remittance receipt and food security through a comprehensive analysis. Using data from the Household Income and Expenditure Survey (HIES) 2022, we developed a food security index incorporating calorie intake, dietary diversity, food expenditure, and the Food Insecurity Experience Scale (FIES) score. Advanced statistical methods, including Seemingly Unrelated Regression (SUR), Zero-Inflated Negative Binomial, linear regression model, inverse-probability-weighting (IPW), and doubly robust method were employed to identify the factors associated with food security and assess the causal effect of remittance earning. Our findings reveal a strong and positive causal effect of remittance receipt on food security. The observed causal effect remained robust against model misspecification and unmeasured confounders, as confirmed through sensitivity analysis. Key factors such as wealth index, residence type, regional differences, household head’s education, and number of earners also influenced food security outcomes. However, the significance of variables like land ownership, household head’s age, and sex varied across measures. This study highlights the transformative role of remittances in reducing food insecurity. Policies that support remittance flows, improve rural infrastructure, and promote skill development and financial literacy can further strengthen their impact.

## Introduction

Global hunger and food insecurity have remained alarmingly high, with 733 million people facing hunger and 2.33 billion experiencing moderate to severe food insecurity in 2023. The prevalence of undernourishment has stagnated since the COVID-19 pandemic, now affecting 9.1% of the global population, compared to 7.5% in 2019 [1]. Stunting also persists as a major concern, with 148 million children under the age of 5 years affected in 2022. These challenges, exacerbated by conflicts and rising food prices, underscore the urgency of achieving the United Nations Sustainable Development Goals (SDGs), particularly Targets 2.1 and 2.2, which focus on eradicating hunger and malnutrition [2].

Bangladesh, a country striving for economic growth amidst escalating threats of climate change, presents a unique context for exploring food security. Climate-induced shocks, such as cyclones, floods, salinity, and droughts, frequently disrupt agricultural production and deepen existing socioeconomic vulnerabilities [3,4]. These shocks disproportionately affect marginalized communities, where food insecurity rates are significantly higher than the national average [5]. At the same time, remittance transfers from Bangladeshi migrants working abroad have become a vital source of livelihood for many households, with potential implications for improving food access and nutritional outcomes [6,7].

Numerous studies have investigated the factors associated with food security. Key findings highlight that higher education levels of household heads, increased livestock income, secure land tenure, larger land sizes, and group memberships significantly enhance household food and nutrition security [8]. Conversely, residence type, women’s educational status, weekly rice consumption, and household characteristics are strongly associated with child hunger [9]. Food insecurity is prevalent in rural districts of Bangladesh, with heightened vulnerability in the northwestern, central-southwestern, and coastal regions [10]. Women’s market access, wealth, vegetable gardens, and literacy correlate positively with dietary diversity, while larger households face a heightened risk of food insecurity [11]. Additionally, mothers in food-insecure households are more likely to have small-sized infants, indicating the critical intersection of maternal health and household food security [12].

The relationship between remittances and food security has been extensively studied, with research highlighting their association with key indicators such as dietary diversity, caloric intake, and food expenditure [6]. Evidence suggests that households receiving remittances tend to have improved access to food and nutrition [13,14]. Additionally, remittances often function as a financial safety net, helping households manage economic instability, natural disasters, and agricultural shocks [15–17]. These benefits are particularly evident in rural areas, where income sources are often unpredictable, and food insecurity is widespread [18,19].

Recent research has extensively explored food security and its associated factors, yet significant gaps remain. In the context of Bangladesh, remittance receipt is a critical source of income for many households, often linked to improved well-being and economic resilience [20]. However, there is limited understanding of its causal effect on food security, especially when food security is measured through different approaches. Studies have employed various measures to assess food security, as there is no universally accepted standard. Some measures emphasize individuals’ experiential perceptions of food insecurity [21–23], while others rely on consumption patterns [6,24–26]. This raises an important question: do observed associations between factors such as remittance receipt and food security differ depending on the measure used? Furthermore, existing studies rarely investigate whether these associations reflect causal effects or simple correlations. To address these gaps, this study has three main objectives: first, to analyze the associations between various factors and food security across different existing measures; second, to develop a comprehensive food security index that integrates these measures; and finally, to investigate whether the observed associations represent causal effects, with a particular focus on remittance receipt.

**Fig 1:**
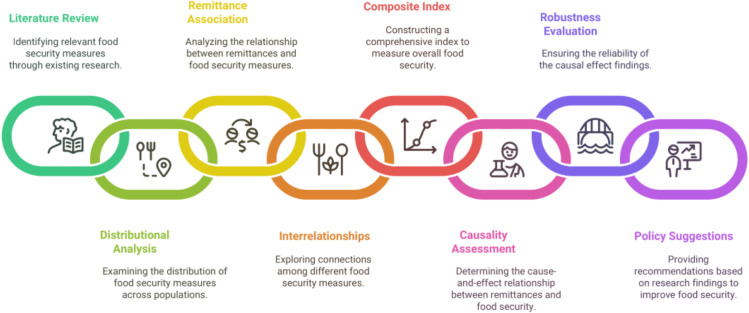
Conceptual framework of the study.

Fig 1 presents the conceptual framework of our study. We begin by identifying the commonly used measures of food security in Bangladesh and examining their distributional characteristics to guide the choice of appropriate statistical methods. Next, we investigate the association between remittances and each individual food security measure. Based on the interrelationships among these measures, we then construct a composite food security index and assess its association with remittances. Finally, we evaluate whether the observed associations are causal and examine the robustness of our findings to potential unmeasured confounding.

## Methods

### Data

We utilized data from the latest round of Household Income and Expenditure Survey (HIES). We use HIES 2022 because it is the first survey wave in Bangladesh to include the Food Security Experience Scale (FIES). This dataset also provides comprehensive information on individual and household income, consumption expenditure, educational attainment, health indicators and various other demographic characteristics. The Bangladesh Bureau of Statistics (BBS) conducted the survey using a two-stage stratified cluster sampling design. The Primary Sampling Unit (PSU) was the Enumeration Area (EA) from the Population and Housing Census of 2022. At the first stage, the required number of PSUs was selected and a complete household listing was carried out in the selected PSUs. Then at the second stage, 20 households were selected randomly from each selected PSU for the interview in the field, resulting in a final sample of 14400 households. In our analysis, we included 13565 households after excluding those with missing values. Further information about the sampling design can be found in the published report of HIES 2022 [27].

#### Outcome variable.

We employ widely recognized measures of food security commonly utilized in the literature. Based on a comprehensive review of existing studies, we adopt the following four measures of food security: the Household Dietary Diversity Score (HDDS) [6,25,26], Per Capita Daily Calorie Intake [6,28], Food Expenditure as a Percentage of Total Expenditure [25], and the FIES score [21–23]. Then a composite index with these indicators were created by applying Principal Component Analysis and retaining the first PC scores.

The Household Dietary Diversity Score is defined as the total number of distinct food groups consumed by a household within a single day. The food items consumed are divided into 12 distinct groups and if a household consumed an item from a specific food group, the household was assigned a value of “1” for that food group and “0” otherwise. So the score had a range of 0 to 12. For each household, HDDS is calculated for 14 days and then averaged to obtain the HDDS per day.

The food items consumed given in units of gram or kg were converted into kcal to obtain the total calorie intake for each household using the food consumption table for Bangladesh [29]. The consumption of food was given for a period of 14 days. Averaging by day and member of each household, the per capita daily calorie intake was calculated.

To calculate the percentage of expenditure on food, the food consumption reported over a 14-day recall period was used to find the household’s monthly food expenditure. Similarly, the monthly expenditure from non-food consumption items were calculated. The percentage of food expenditure was then estimated as a proportion of each household’s total monthly expenditure. The formula is given below:


$$\text{Food Expenditure (\%)}=\frac{\text{Total expenditure on food}}{\text{Total expenditure}}\times 100.$$

For calculating the FIES score, the eight questions that were used in the HIES survey were during the last 12 months if any of the household members was worried about not having enough food, unable to eat healthy and nutritious food, ate only a few kinds of food, had to skip a meal, ate less than should have eaten, household ran out of food, was hungry but did not eat and went without eating for a whole day due to a lack of money or other resources. The FIES score is then calculated by summing the affirmative responses to the eight questions.

#### Exposure variable.

The exposure variable in this study is the remittance receiving status of households which is a binary variable with two levels: “yes” if the household received any remittances and “no” if not received any remittances either within Bangladesh or abroad over the last 2 years.

#### Control variables.

The control variables include age of the household head, education of the household head (no education, primary, secondary and higher), sex of the household head (male and female), total land owned, received assistance from social safety net programs in the past 12 months (yes, no), presence of chronic disease of any member of household (yes, no), number of household members, dependency ratio, number of earners, area of residence (urban and rural), division (Rangpur, Barishal, Chattogram, Dhaka, Khulna, Mymensingh, Rajshahi and Sylhet), and wealth index (poorest, poorer, middle, richer and richest). The wealth index is created by applying PCA to the appropriate variables and dividing the first PC scores into five groups.

### Statistical analysis

#### Seemingly unrelated regression model.

Food security measures are often correlated with one another, and these correlations should be accounted for when identifying associated factors. The Seemingly Unrelated Regression (SUR) model provides a framework to address this issue by explicitly considering the correlations among the measures.

The SUR is a structural equation technique that employs a two-stage estimation procedure to simultaneously analyze all outcome variables, while accounting for their correlated error structures. This method was first introduced by [30]. Let *Y*_*k*_ represents the *k^th^* dependent variable and *X*_*j*_ represents the *j^th^* independent variable. Consider the following system of linear equations:


$$Y_{1i}=\beta_{10}+\beta_{11}X_{1i}+\ldots +\beta_{1p}X_{pi}+\epsilon_{1i}\;Y_{2i}=\beta_{20}+\beta_{21}X_{1i}+\ldots +\beta_{2p}X_{pi}+\epsilon_{2i}\;\vdots \;Y_{Ki}=\beta_{K0}+\beta_{K1}X_{1i}+\ldots +\beta_{Kp}X_{pi}+\epsilon_{Ki},$$

where $\epsilon_{1},\epsilon_{2},\ldots ,\epsilon_{K}$ are the error terms associated with the *K* linear equations and $\beta_{ij}$ is the coefficient of the *j^th^* covariate on the *i^th^* equation. Here, we assume the same set of covariates for each equation, although this is not mandatory for SUR models.

To estimate the parameters of the SUR model, a two-step procedure is followed. In the first step, ordinary least squares (OLS) is employed to estimate the covariance of error terms across all outcomes. In the second step, feasible generalized least squares (FGLS) are applied to the system of equations to address the correlated error structures. Consequently, the SUR approach is superior to the equation-by-equation OLS model, as it produces more robust parameter estimates for coefficients, standard errors, and covariances, ultimately leading to more efficient inference [31].

#### Zero inflated negative binomial regression model.

The Zero-Inflated Negative Binomial (ZINB) is a model designed for count data that exhibit overdispersion and an excess of zero counts than what is expected under a standard negative binomial distribution. A ZINB regression model for the response variable $Y_{i}\;(i=1,\ldots ,n)$ can be defined by the following probability mass function:


$$\mathrm{Pr}(Y_{i}=y_{i})=\left\{ \begin{array}{cc} p_{i}+(1-p_{i}){\left(\frac{\phi }{\mu_{i}+\phi }\right)}^{\phi }, & \text{if }y_{i}=0\\ (1-p_{i})\frac{\Gamma (\phi +y_{i})}{\Gamma (y_{i}+1)\Gamma (\phi )}{\left(\frac{\mu_{i}}{\mu_{i}+\phi }\right)}^{y_{i}}{\left(\frac{\phi }{\mu_{i}+\phi }\right)}^{\phi }, & \text{if }y_{i}>0, \end{array}\right.$$

where $0\leq p_{i}\leq 1$, $\mu_{i}\geq 0$, $\phi^{-1}$ is the dispersion parameter with ϕ>0, and Γ(·) is the gamma function.

The parameters $\mu_{i}$ and *p*_*i*_ are determined by the explanatory variable vectors ${𝐱}_{i}$ and ${𝐳}_{i}$, respectively. The models are assumed as follows:


$$\mu_{i}=\exp\left({𝐱}_{i}^{T}\beta \right)\;\text{and}\;p_{i}=\frac{\exp\left({𝐳}_{i}^{T}\gamma \right)}{1+\exp\left({𝐳}_{i}^{T}\gamma \right)},$$

where $\beta =(\beta_{1},\ldots ,\beta_{p}{)}^{T}$ and $\gamma =(\gamma_{1},\ldots ,\gamma_{q}{)}^{T}$ are unknown parameters. However, similar results can be derived for other link functions. The parameters are typically estimated by maximizing the likelihood function using numerical optimization methods [32].

While negative binomial models are appropriate for overdispersed count data, binomial regression is generally preferred when the outcome is bounded (e.g., restricted to values between 0 and 8). In particular, when the response variable represents the sum of several binary indicators, the resulting count data is inherently bounded. In such cases, count models such as Poisson or negative binomial regression may still be employed, especially when the probability of success in each trial (i.e., the probability of a “yes” response to each question) is low [33]. Before employing a negative binomial model, a test for overdispersion should be performed, which is detailed in the paper by Cameron and Trivedi [34].

#### Principal component analysis.

Principal Component Analysis (PCA), a widely used multivariate technique is used to reduce the dimensionality of a dataset comprising numerous correlated variables while preserving as much of the original variation as possible. This is accomplished by transforming the data into a new set of variables called Principal Components (PCs) which are the linear combinations of the original variables. Let X be an n×p data matrix with *p n*-dimensional vectors $x_{1}$, $x_{2}$, ... , $x_{p}$ where the *j*th column represents the vector $x_{j}$, which contains the observations for the *j*th variable. The PCs are the linear combinations $\sum_{j=1}^{p}a_{j}x_{j}=Xa$ where a represents a vector of constants *a*_1_, *a*_2_, ..., *a*_*p*_. The values of *a*_*j*_ are obtained from the eigenvectors of the covariance or correlation matrix of the dataset. The PCs are uncorrelated, orthogonal and ranked in such a way that the first few components capture the majority of the variation in the original dataset. The elements of the linear combinations are referred to as PC scores. These scores represent the transformed values that each individual achieves on a specific principal component. PCA is frequently used for creating different indices [35].

#### Causal inference.

Causal inference techniques are important because they can demonstrate a cause-effect relationship between variables that are not influenced by confounders. These methods can be particularly useful in establishing an unconfounded relationship between remittance receipts and household food security.

A causal effect denotes the impact that one variable (the cause or treatment) exerts on another variable (the outcome), thereby establishing a cause-and-effect relationship. Unlike associations or correlations, a causal effect suggests that variations in the cause will directly result in changes in the outcome. The counterfactual framework is a foundational approach in causal inference, focusing on comparing potential outcomes under different treatments or conditions for the same individual or unit [36]. This framework poses the question, “What would the outcome *Y* have been if the treatment or exposure *Z* had taken a different value?" Let *Y*^*z* = 1^ and *Y*^*z* = 0^ represent the two potential outcomes under the respective treatment levels. However, for each subject, only one of these potential outcomes is observed. Consequently, the observed outcome is expressed as


$$Y=ZY^{z=1}+(1-Z)Y^{z=0}.$$

In causal inference, the goal is often to estimate the average treatment effect (ATE), which represents the average difference in outcomes if everyone received the treatment versus if no one did. Mathematically, the ATE is defined as:


$$\tau =E(Y^{z=1}-Y^{z=0}).$$

In general, three primary approaches are used to estimate the ATE: the exposure modeling approach, the outcome modeling approach, and the doubly robust estimator.

The exposure modeling approach, also known as the inverse probability weighting (IPW) method, involves estimating the probability of receiving the treatment given covariates, known as the propensity score. Let e(X)=Pr(Z=1∣X) denote the propensity score, and X denote the vector of covariates. Once the propensity scores are estimated, the IPW method estimates the ATE using the following formula [37]:


$${\hat{\tau }}_{ipw}=\frac{1}{n}\sum_{i=1}^{n}\frac{Z_{i}Y_{i}}{\hat{e}\left(X_{i}\right)}-\frac{1}{n}\sum_{i=1}^{n}\frac{\left(1-Z_{i}\right)Y_{i}}{1-\hat{e}\left(X_{i}\right)}.$$

In the outcome modeling approach, also known as g-computation or g-formula, we directly model the outcome *Y* as a function of treatment *Z* and covariates X. After specifying the model E(Y∣Z,X), the ATE is estimated as follows [38]:


$${\hat{\tau }}_{gcomp}=\frac{1}{n}\sum_{l}\hat{E}(Y|Z=1,X)-\frac{1}{n}\sum_{l}\hat{E}(Y|Z=0,X).$$

The doubly robust estimator combines both the exposure and outcome modeling approaches, offering protection against model misspecification. This approach leverages both the propensity score and outcome models, and it is consistent if at least one of these models is correctly specified [39]. The doubly robust estimator of ATE can be represented as


$${\hat{\tau }}_{DR}=\frac{1}{n}\sum_{i=1}^{n}\left[\frac{Z_{i}Y_{i}}{\hat{e}(X_{i})}-\frac{Z_{i}-\hat{e}(X_{i})}{\hat{e}(X_{i})}\hat{E}(Y_{i}|Z_{i}=1,X_{i})\right]\;\;-\frac{1}{n}\sum_{i=1}^{n}\left[\frac{(1-Z_{i})Y_{i}}{1-\hat{e}(X_{i})}-\frac{\hat{e}(X_{i})-Z_{i}}{1-\hat{e}(X_{i})}\hat{E}(Y_{i}|Z_{i}=0,X_{i})\right].$$

## Results

All analyses for this study were conducted using R (version 4.3.1). The ggplot2 package was utilized to create visualizations, while the systemfit package was employed to fit the SUR models.

### Distributional characteristics of food security measures

**Fig 2:**
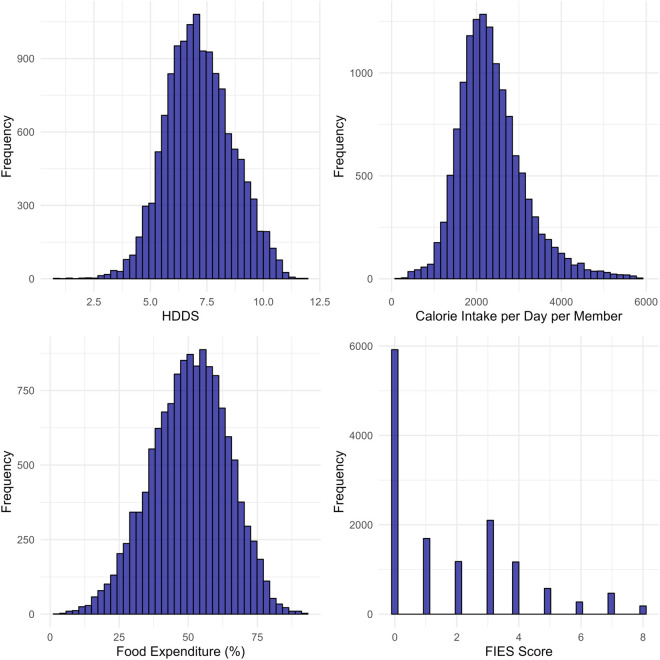
Histogram of each food security measures separately.

Fig 2 demonstrates histograms for the four distinct food security measures, providing insights into their distributional characteristics. The first three food security measures (household dietary diversity score, per capita daily calorie intake and percentage of expenditure on food) roughly exhibit a normal distribution although a few outliers are observed. The FIES score is a count variable ranging from 0 to 8. However, the large number of zeros makes it unclear if a Poisson model is appropriate, as the excess zeros could lead to over-dispersion. Descriptive statistics for all the variables used in this study are provided in the Supplementary information (see S1 Table).

### Trends in food security measures by remittance status

**Fig 3:**
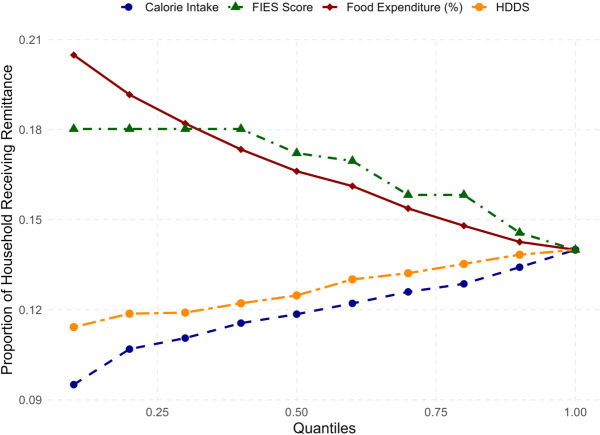
Line chart of proportion of households receiving remittance by quantile of food security measures.

All four food security measures demonstrate a trend, either upward or downward with remittance as depicted in Fig 3. Among them, the HDDS and per capita daily calorie intake reflect the extent of food security, both increasing with the increase of proportion of households receiving remittance. Conversely, the percentage of expenditure on food and FIES score rises with the decrease of proportion of households receiving remittance, indicating the state of food insecurity.

In households classified within the lowest 10% of the per capita daily calorie intake, approximately 9% receive remittances. This proportion increases to 12% when considering the group comprising the lowest 50% of households by calorie intake. The proportion gradually increases for higher quantile groups and reaches 14% when the entire sample is taken. For the the lowest 10% of the HDDS group, the proportion of households receiving remittance is below 12% which goes above 12% when the lowest 50% households are considered. Similar to the previous case, the proportion becomes 14% when all households are considered.

The proportion of households receiving remittance is 18% in the 0.1 quantile or lowest 10% of the FIES score group. This proportion remains stable until the 0.4 quantile and then begins to decline, eventually settling at 14% when considering the entire dataset. Regarding the percentage of expenditure on food, slightly less than 21% of households in the lowest 10% receive remittances. This figure gradually declines, surpassing 16% when considering the lowest 50% of households. As we move towards higher quantile groups, the proportion continues to drop and ultimately falls to 14% for all households. The four lines meet at 0.14 representing the overall proportion of households receiving remittance is 14%.

### Construction of the food security index

**Fig 4:**
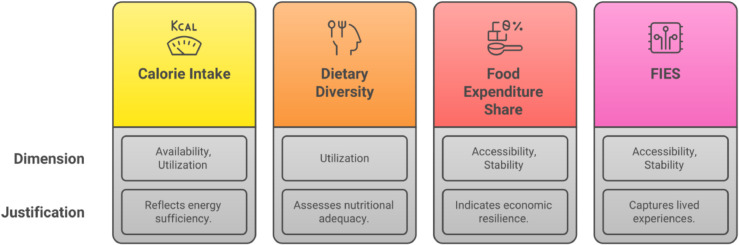
Components of food security index and their dimensions.

To construct the food security index, three key considerations were taken into account: (1) the commonly used measures of food security in Bangladesh; (2) the internationally recognized dimensions of food security; and (3) the statistical feasibility of combining the selected measures. According to the Food and Agriculture Organization (FAO), food security comprises four dimensions: availability, accessibility, utilization, and stability [40]. The authors reviewed commonly used food security indicators in the context of Bangladesh and mapped each indicator to the corresponding FAO-defined dimension. Fig 4 presents the conceptual framework for the food security index, illustrating the classification of indicators and the rationale for their inclusion.

**Fig 5:**
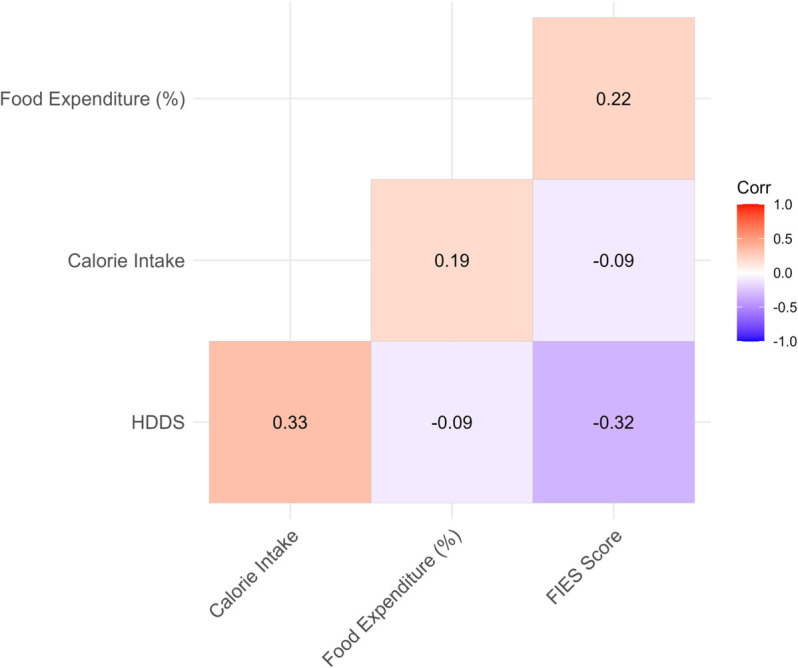
Visualizations related to food security: Correlation heatmap of food security measures.

The correlation heatmap of the food security measures is presented in Fig 5. A strong positive correlation of 0.33 exists between HDDS and calorie intake, suggesting that as HDDS increases, calorie intake also tends to increase. Conversely, both food expenditure (%) and the FIES score exhibit negative correlations with HDDS. Specifically, these relationships indicate that increases in HDDS are associated with decreases in both food expenditure (%) and FIES scores. Additionally, the correlation between the FIES score and food expenditure is weakly positive, with a value of 0.22. There is also a positive association between calorie intake and food expenditure (%), with a correlation value of 0.19. A weak negative association (-0.09) is observed between the FIES score and calorie intake. This suggests that higher levels of calorie intake are associated with lower FIES scores. The high correlation among food security measures motivates the use of multivariate techniques, such as PCA, to construct a composite food security index.

The first principal component had an eigenvalue of 1.52 (standard deviation = 1.23) and explained 38% of the total variance. The second, third, and fourth components had eigenvalues of 1.24, 0.67, and 0.57, explaining 31%, 17%, and 14% of the variance, respectively. Since the objective was to derive a univariate measure of food security, only the first principal component was retained, and its scores were used as the composite food security index. This index is a linear combination of the individual food security measures that captures the maximum variability. The food security index can be represented as


FoodSecurityIndex=0.664×HDDS+0.453×Calorie Intake+0.182×(100−Food Expenditure(%))+0.567×(8−FIES Score).

**Fig 6:**
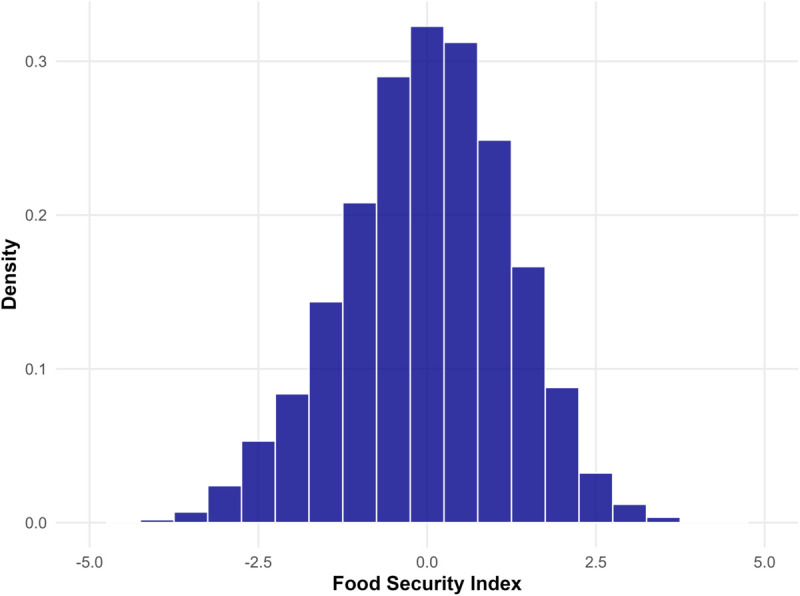
Histogram of the developed food security index.

Fig 6 displays the histogram of the food security index. The food security index approximately follows a normal distribution as shown by the bell shaped histogram. The higher the index value, the more food-secure a household is. The values of the index roughly varies between –3 to 3.

### Regression analysis: Association of remittance and food security measures

Table 1 portrays the results from the SUR and Zero Inflated Negative Binomial Regression models. The use of the SUR model is motivated by the observed high correlation among the food security measures: HDDS, calorie intake, and food expenditure (%), as well as their bell-shaped distributions. A test for overdispersion for the FIES score yielded a p-value of 0.159. While this does not reach statistical significance, the data suggest a potential overdispersion. The data also shows a low probability of “yes” response in the FIES questions, motivating the use of a ZINB model.

**Table 1:** Combined results from seemingly unrelated, zero inflated negative binomial, and linear regression.

	SUR						ZINB		LR	
	HDDS		Calorie Intake		Food Exp(%)		FIES Score		FSI	
**Variables**	Est.	p-val	Est.	p-val	Est.	p-val	IRR	p-val	Est.	p-val
**Received Remittance**										
No										
Yes	0.109	0.002	150.251	<0.001	–2.117	<0.001	0.736	<0.001	0.342	<0.001
**Area of Residence**										
Rural										
Urban	0.393	<0.001	–144.335	<0.001	–1.983	<0.001	1.027	0.080	0.077	<0.001
**Household Head Sex**										
Male										
Female	–0.173	<0.001	5.419	0.817	–2.419	<0.001	1.067	0.007	–0.090	0.004
**Household Head Age**	0.002	0.019	6.858	<0.001	–0.015	0.082	0.997	<0.001	0.007	<0.001
**Total Land**	0.002	0.471	4.694	0.002	–0.001	0.979	0.974	<0.001	0.008	<0.001
**Wealth Index**										
Poorest										
Poorer	0.216	<0.001	98.213	<0.001	–1.732	<0.001	0.881	<0.001	0.317	<0.001
Middle	0.406	<0.001	142.303	<0.001	–3.022	<0.001	0.790	<0.001	0.529	<0.001
Richer	0.656	<0.001	214.774	<0.001	–5.455	<0.001	0.708	<0.001	0.804	<0.001
Richest	1.016	<0.001	257.438	<0.001	–10.693	<0.001	0.540	<0.001	1.182	<0.001
**Safety Net**										
No										
Yes	–0.081	<0.001	12.446	0.390	1.218	<0.001	1.067	<0.001	–0.127	<0.001
**Chronic Disease**										
No										
Yes	0.099	<0.001	33.929	0.031	–3.398	<0.001	1.053	0.002	0.084	<0.001
**Division**										
Rangpur										
Barishal	0.079	0.078	–108.147	<0.001	2.291	<0.001	0.891	<0.001	0.055	0.132
Chattogram	0.332	<0.001	110.317	<0.001	3.147	<0.001	1.004	0.892	0.153	<0.001
Dhaka	0.899	<0.001	65.222	0.015	–4.007	<0.001	0.919	0.005	0.627	<0.001
Khulna	0.710	<0.001	165.985	<0.001	2.153	<0.001	0.891	<0.001	0.422	<0.001
Mymensingh	0.809	<0.001	226.544	<0.001	1.366	0.001	0.795	<0.001	0.661	<0.001
Rajshahi	0.507	<0.001	168.566	<0.001	0.162	0.698	0.834	<0.001	0.483	<0.001
Sylhet	0.728	<0.001	325.613	<0.001	4.708	<0.001	1.038	0.169	0.392	<0.001
**Household Head Education**										
No education										
Primary	0.189	<0.001	–6.364	0.733	–1.690	<0.001	0.919	<0.001	0.183	<0.001
Secondary	0.324	<0.001	9.766	0.608	–3.365	<0.001	0.833	<0.001	0.403	<0.001
Higher	0.571	<0.001	–58.080	0.016	–7.781	<0.001	0.760	<0.001	0.659	<0.001
**Dependency Ratio**	0.001	0.016	–1.547	<0.001	0.011	<0.001	1.001	<0.001	–0.001	<0.001
**Household Members**	0.099	<0.001	–194.051	<0.001	0.107	0.158	0.966	<0.001	–0.033	<0.001
**Earners**	0.097	<0.001	60.176	<0.001	–0.317	0.041	0.957	<0.001	0.110	<0.001
**(Intercept)**	5.029	<0.001	2699.103	<0.001	59.709	<0.001	5.192	<0.001	–1.532	<0.001

Table notes: SUR = Seemingly Unrelated Regression; ZINB = Zero-Inflated Negative Binomial; LR = Linear Regression; HDDS = Household Dietary Diversity Score; FIES = Food Insecurity Experience Scale; FSI = Food Security Index; Food Exp(%) = Share of household food expenditure as a percentage of total expenditure; Est. = Estimated coefficient; IRR = Incidence Rate Ratio; p-val = p-value.

The SUR model reveals the association between remittance and the three food security measures HDDS, calorie intake and food expenditure (%) separately. For households receiving remittances, the HDDS is on average 0.109 points higher compared to households that do not receive remittances, keeping the other variables constant. Likewise, the per capita daily caloric intake in remittance-receiving households exceeds that of non-receiving households by 150.251 kcal on average. On the contrary, food expenditure as a percentage of the total expenditure shows a negative association, with remittance-receiving households spending, on average, 2.117% less of their total expenditure on food relative to those that do not receive remittance. The ZINB regression model shows an incidence rate ratio (IRR) of 0.736, indicating that the rate of “yes” answers to the FIES questions is about 73.6% of the rate for subjects who did not receive remittances. Specifically, receiving remittance is associated with a 26.4% reduction in the expected counts of “yes” answers compared to those who did not receive remittances.

The analysis of the four regression models reveals several significant associations between control variables and measures of food security. Notably, the area of residence is significantly related to the HDDS, calorie intake, and food expenditure percentage, but shows no significant association with the FIES score. Additionally, the sex of the household head significantly influences the HDDS, food expenditure percentage, and FIES score, while no significant relationship is found with calorie intake. The age of the household head demonstrates a significant association with HDDS, calorie intake, and the FIES score, though it does not significantly correlate with food expenditure percentage. Furthermore, the total land owned by the household is associated with calorie intake and the FIES score, but this relationship is not significant for the other two food security measures at the 5% significance level. The Wealth Index emerges as significantly associated with all four measures of food security. Similarly, receiving assistance from any safety net program is linked to all measures except for calorie intake. Other variables, including the presence of chronic disease, division, household head’s education, dependency ratio, number of household members, and number of earners, are also significantly associated with all four food security measures.

Regional disparities play a crucial role in shaping food security outcomes. Table 1 presents how the different food security measures, as well as the overall food security index, vary across divisions in Bangladesh. Rangpur division, widely recognized as one of the most poverty-prone areas of the country, is taken as the reference group. Relative to Rangpur, all divisions exhibit higher dietary diversity, as reflected by the positive coefficients. Calorie intake is also higher in other divisions, with Barisal being the only division that records significantly lower intake than Rangpur. Dhaka stands out for having the lowest share of food expenditure relative to total household expenditure—on average, 4% less than Rangpur. The FIES score and the composite food security index are also higher in all divisions compared to Rangpur. Taken together, the results reaffirm Rangpur’s status as the most food-insecure division. The coefficient for Barishal is small (0.055) for the food security index but statistically insignificant.

The results of the linear regression analysis on the developed food security index are also presented in Table 1. Households receiving remittances exhibit an average increase of 0.342 in the index compared to those not receiving remittances, after adjusting for other control variables. This association between remittances and the food security index is statistically significant, as indicated by a p-value of less than 0.001. Furthermore, households located in urban areas have an average index value that is 0.077 higher than that of rural households, when controlling for other variables. The analysis also highlights that additional control variables, including the age of the household head, total land, safety net participation and the presence of chronic diseases are statistically significant. Moreover, the wealth index, the education level of the household head, division, the dependency ratio, and the number of earners are significantly associated with the food security index.

### Causal effect of remittance on the food security index

#### Determining confounders.

To establish a causal effect, it is essential to identify potential confounders. A variable qualifies as a suitable confounder if it influences both the treatment and the outcome. Table 2 presents the selected confounders along with the rationale for their inclusion.

**Table 2:** Reason of inclusion of potential confounders.

Potential Confounder	Reason of Inclusion
Area of Residence	The food security level is usually lower in rural areas and the possibility of receiving remittances differs in urban and rural areas.
Household Head Age	Households with older household heads may have more food security because of experience, and likelihood of receiving remittances may increase since families support older relatives.
Total Land	Land ownership affects food security through food production, and larger landholdings may also help households send more migrants abroad, thereby increasing remittances.
Wealth Index	Wealthy households are more likely to be food secure and less dependent on remittances.
Safety Net	Recipients of safety nets are often poorer, affecting food security, and tend to rely more on remittances.
Chronic Disease	Chronic illness can reduce food security and potentially increase reliance on remittances for medical expenses.
Household Head Education	Higher levels of education may increase income or knowledge of nutrition, positively influencing food security. Additionally, more educated individuals might be more likely to migrate or earn higher wages abroad.
Dependency Ratio	Households with higher dependency ratios may have lower food security and are more likely to receive remittances.
Household Members	Larger households may have different food security levels due to higher consumption and may also have increased chances of receiving remittances due to greater need.
Earners	More earners could improve food security and reduce dependence on remittances.

Table notes: This table presents the rationale for including each potential confounder in the analysis.

#### Assessing balance in the confounders.

**Fig 7:**
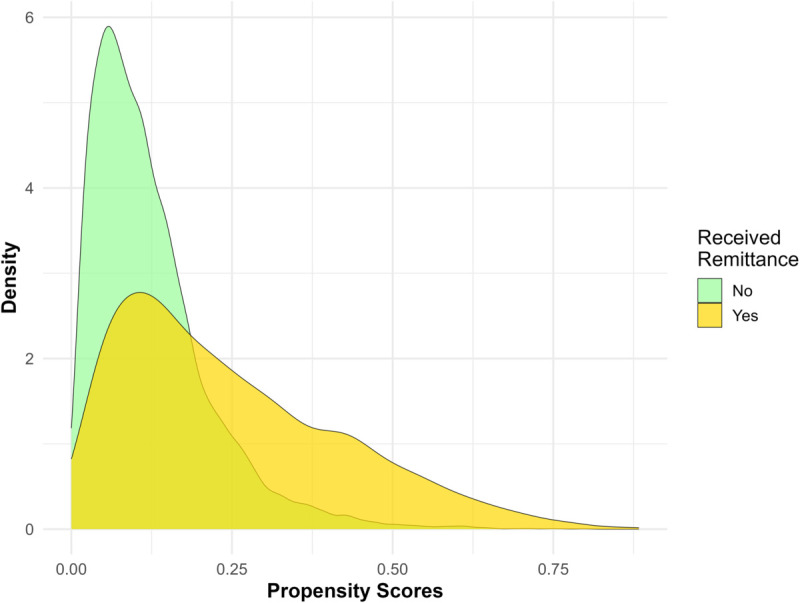
Distribution of the estimated propensity scores across the two remittance receiving groups.

**Fig 8:**
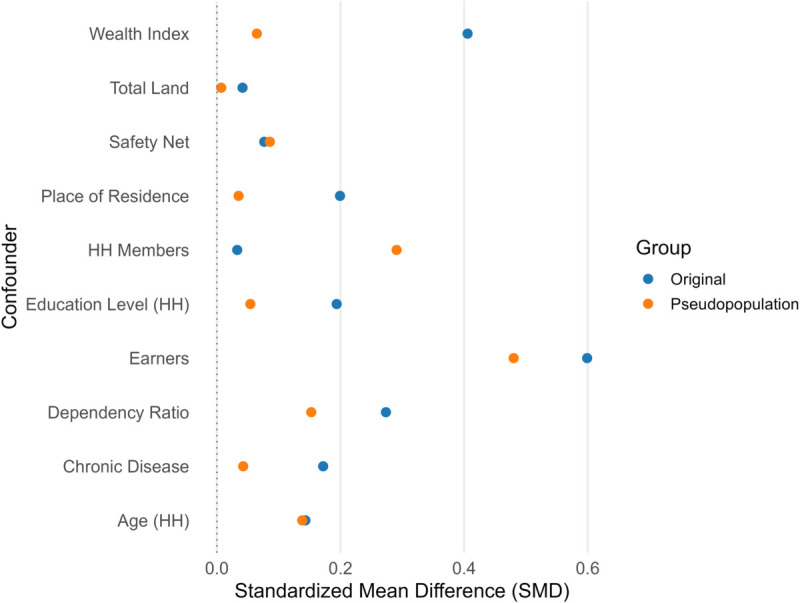
Standardized mean difference for each confounder before and after adjustment.

Fig 7 illustrates the distribution of estimated propensity scores for the groups receiving and not receiving remittances. A greater overlap between the two distributions indicates a better balance in the observed confounders across the treatment groups. The figure reveals a substantial overlap in the distributions, providing evidence of effective balance achieved between the groups. To further assess whether propensity score weighting helps to achieve good balance in the observed covariates, we calculate the standardized mean difference (SMD) for each confounder. Fig 8 shows the estimated SMD for each confounder before and after weighting. This figure reveals that weighting can substantially reduce the confounding bias as evident from the lower SMD in the weighted pseudo-population.

#### Estimated average treatment effect.

The ATE is estimated using three different estimators: IPW estimator, the g-computation estimator, and the doubly robust estimator, which is presented in Table 3. The estimated ATE of remittances on the food security index is 0.365 ($\hat{ATE}$ = 0.365, 95% *CI*: (0.324, 0.405)). This suggests that if every individual in the population received remittances, the average food security score would be 0.365 units higher than if no individuals received remittances. Similar estimates were obtained using the other methods: the g-computation method yielded an ATE of 0.323 ($\hat{ATE}$ = 0.323, 95% *CI*: (0.269, 0.376)), while the doubly robust estimator provided an estimated ATE of 0.284 ($\hat{ATE}$ = 0.284, 95% *CI*: (0.163, 0.404)). For the g-computation method and the doubly robust method, we utilized bootstrapping to obtain the estimated standard error and the 95% confidence interval. The consistency of the estimates across all three methods indicates that both the treatment and outcome models are likely well-specified.

**Table 3:** Estimated ATE of remittance on food security index using different methods and corresponding 95% confidence interval.

Method	Estimated ATE	Std. Error	95% CI
IPW	0.365	0.021	(0.324, 0.405)
g-computation	0.323	0.027	(0.269, 0.376)
Doubly Robust	0.284	0.061	(0.164, 0.404)

Table notes: This table reports the estimated average treatment effect (ATE) of remittance on the food security index using different estimation methods. Standard errors are shown in column 3, and column 4 provides the corresponding 95% confidence intervals.

We further examine the causal effects of different types of remittances (internal and international) on the constructed food security index. Our analysis indicates that international remittances exert a stronger positive impact on food security compared to internal remittances. Detailed results are provided in the Supporting information (see S2 Table). We also examine the causal effect of remittance receipts on each individual food security measure separately. The corresponding results are provided in the Supporting information (see S3 Table).

#### Sensitivity analysis.

**Fig 9:**
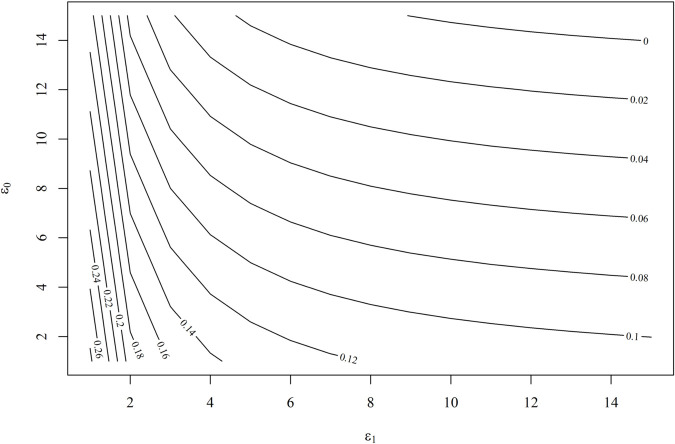
Sensitivity analysis for the estimated ATE using doubly robust method.

**Fig 10:**
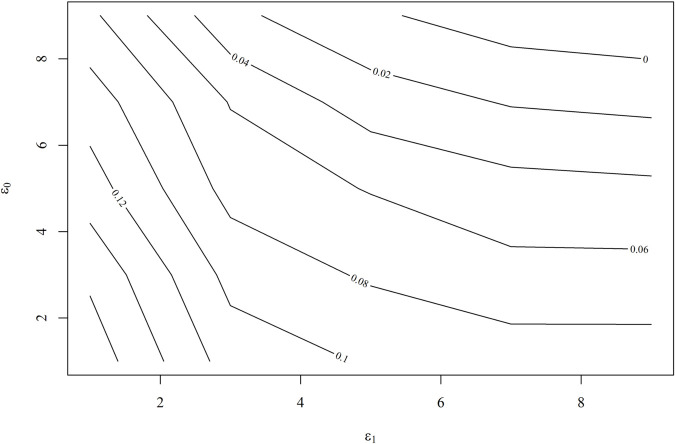
Sensitivity analysis for the lower limit of the confidence interval for ATE using doubly robust method.

To assess the strength of an unmeasured confounder necessary to influence our estimated causal effects, we conduct a sensitivity analysis using the doubly robust method proposed by Lu and Ding [41]. Fig 9 illustrates that, to nullify the estimated causal effect of remittance receipt on the food security index, very high values of the sensitivity parameters $\epsilon_{0}$ and $\epsilon_{1}$ are required. We selected a range of values for the sensitivity parameters $\epsilon_{1}$ and $\epsilon_{0}$, spanning from 1 to 15. The estimated causal effect approaches zero when $\epsilon_{1}$ exceeds 10 and $\epsilon_{0}$ exceeds 14. Such strong unmeasured confounders are unlikely to be overlooked among the observed confounders, suggesting that the validity of the estimated causal effect is very high. Similarly, Fig 10 shows the result from the sensitivity analysis for the lower limit of the 95% confidence interval for the causal effect. The results indicate that for the observed causal effect of remittances on the food security index to become insignificant, the sensitivity parameter $\epsilon_{1}$ must exceed 8, with $\epsilon_{0}$ also needing to surpass 8. Therefore, the likelihood of overlooking confounders of this magnitude is very low.

## Discussion

Despite advancements in economic growth and poverty reduction, food insecurity remains a significant issue in Bangladesh, driven by climate-related shocks and socioeconomic vulnerabilities. This study develops a comprehensive food security index integrating standard measures and uses advanced statistical models—Seemingly Unrelated Regression (SUR), Zero-Inflated Poisson, and linear regression—to analyze the factors influencing food security. Key determinants include remittance receipt, residence area, wealth index, division, household head’s education, and number of earners, while the significance of variables like land ownership, household head’s age, and sex varies across food security measures.

This study uses data from the HIES 2022, which, for the first time, includes information on food insecurity measured through the FIES developed by the FAO [42]. In addition to this new scale, the study incorporates traditional measures of food security. These include food expenditure, dietary diversity, and calorie intake, which are commonly employed in the literature [28,43,44]. Using these diverse measures, the study develops a food security index that captures their combined effects.

The study reveals a positive association between remittance receipt and all the measures of food security except food expenditure as a percentage of overall expenditure. This aligns with the findings of Raihan et al. [45], which reported a similar association between remittances and consumption expenditure. This result is further supported by Szabo et al. [6], who observed positive associations between remittance receipt, diet diversity, and calorie intake. The negative association between remittance earning and food expenditure suggests that although remittance-receiving households may spend more on food in absolute terms, the share of food in their total expenditure declines. This is consistent with Engel’s Law, a well-known economic principle, stating that share of food in total expenditure is inversely related to the household’s income and wealthier households allocate relatively more spending to non-food items [46]. This law probably also explains the negative coefficient for Dhaka in relation to Barishal on share of expenditure on food measure. The average income of households is higher in Dhaka, possibly leading to lower share of expenditure on food and higher share on non-food items.

In addition to remittance earning, factors such as the wealth index, the education level of the household head, and the type of residence were found to be significantly associated with all measures of food security, consistent with the findings of Szabo et al. and Haque et al. [6,9]. The number of household members also showed significant associations with HDDS, calorie intake, and the FIES score, corroborating the results of Harris-Fry et al. [11].

This study shows that remittance receipt has a positive causal effect on food security index in Bangladesh, providing evidence that the positive association observed by Szabo et al. [6] is, in fact, a causal effect. The observed strong causal effect of remittance on food security further supports the recommendations of Ahmed et al. [47] to encourage remittance through policy initiatives to ensure long-term food security in Bangladesh. By validating the causal effect using different methods and the sensitivity analysis procedures suggested by Lu and Ding [41], the estimated causal effect is further strengthened.

The strong causal effect of remittance on food security observed in this study is consistent with findings from other contexts. For instance, Mabrouk and Mekni [48] highlight the critical role of remittances in enhancing food security, improving food access and stability, and ensuring better food quality in African countries. Similarly, studies from Nepal and Mexico [49,50] demonstrate that households receiving international remittances were significantly more food secure compared to those that did not receive such remittances. Further support for our findings comes from cross-country analyses, such as Subramaniam et al. [51], which examined 51 developing countries and found that higher remittance inflows are associated with increased levels of food supply. These studies collectively reinforce the importance of remittances as a key driver of food security in Bangladesh and beyond.

In recent years, remittance transfers from Bangladeshi migrants working abroad have emerged as a critical source of livelihood for many households, with notable potential to enhance food access and improve nutritional outcomes [20]. Thus, a central objective of this study was to assess the causal effect of remittance receipt on household food security in Bangladesh. This study highlights a strong and positive causal effect of remittances on food security, which proves to be robust against model misspecification and unmeasured confounders. Identifying the causal pathways to food security is crucial as it provides evidence of direct effects unconfounded by other factors, thereby establishing robust cause-and-effect relationships.

These findings hold important policy implications for reducing food insecurity in Bangladesh. Policies should prioritize reducing remittance transaction costs, improving financial infrastructure, and promoting secure remittance channels. Leveraging remittances to strengthen rural infrastructure, agricultural development, and market access can further reduce food insecurity. Additionally, financial literacy programs targeting rural remittance-receiving households can encourage productive investments in agriculture and small businesses, promoting long-term food security.

## Supporting information

S1 Table Descriptive statistics. (PDF)

S2 Table Estimated ATE of types of remittance on food security index using IPW and corresponding 95% confidence interval. (PDF)

S3 Table Estimated ATE of overall remittance on food security indicators using IPW and corresponding 95% confidence interval. (PDF)
