# Supplementary material for: Are remittances a buffer against food insecurity? Lessons from a national survey in Bangladesh
Source: PLoS One. 2025 Oct 17;20(10):e0334391. doi: 10.1371/journal.pone.0334391 (PMC12533901; doi:10.1371/journal.pone.0334391)
Supplement: S1 Table — (PDF) [file pone.0334391.s001.pdf]

Descriptive Statistics for Categorical Variables

Variable	Category	Frequency	Percentage
**Remittance**	No	11,666	86.0%
	Yes	1,899	14.0%
**Place of Residence**	Rural	6,761	49.9%
	Urban	6,804	50.1%
**Sex of Household Head**	Male	12,086	89.1%
	Female	1,479	10.9%
**Wealth Index**	Poorest	2,952	21.7%
	Poorer	2,410	17.8%
	Middle	2,783	20.5%
	Richer	2,743	20.2%
	Richest	2,677	19.8%
**Social Safety Net**	No	8,311	61.3%
	Yes	5,254	38.7%
**Chronic Disease**	No	3,681	27.4%
	Yes	9,884	72.6%
**Division**	Rangpur	1,696	12.5%
	Barishal	1,696	12.5%
	Chattogram	1,693	12.5%
	Dhaka	1,700	12.5%
	Khulna	1,700	12.5%
	Mymensingh	1,651	12.2%
	Rajshahi	1,733	12.8%
	Sylhet	1,696	12.5%
**Education Level**	No education	3,938	29.0%
	Primary	3,400	25.1%
	Secondary	3,947	29.1%
	Higher	2,280	16.8%

Descriptive Statistics for Continuous Variables

Variable	Mean	SD
Household Diet Diversity	7.17	1.47
Calorie intake (per day per member)	2384.41	859.49
Food expenditure (%)	50.52	13.82
FIES Score	1.79	2.09
Age of household head (years)	46.54	13.24
Total land	0.66	4.40
Dependency ratio	62.72	58.26
Household members	4.39	1.73
Number of earners	1.37	0.82
