# Supplementary material for: Are remittances a buffer against food insecurity? Lessons from a national survey in Bangladesh
Source: PLoS One. 2025 Oct 17;20(10):e0334391. doi: 10.1371/journal.pone.0334391 (PMC12533901; doi:10.1371/journal.pone.0334391)
Supplement: S2 Table — (PDF) [file pone.0334391.s002.pdf]

S2 Table: Estimated ATE of Types of Remittance on Food Security Index using IPW and corresponding 95% Confidence Interval

Remittance Type	Estimated ATE	Std. Error	95% CI
Internal	0.085	0.021	(0.043, 0.127)
International	0.565	0.019	(0.527, 0.604)
