# Supplementary material for: Are remittances a buffer against food insecurity? Lessons from a national survey in Bangladesh
Source: PLoS One. 2025 Oct 17;20(10):e0334391. doi: 10.1371/journal.pone.0334391 (PMC12533901; doi:10.1371/journal.pone.0334391)
Supplement: S3 Table — (PDF) [file pone.0334391.s003.pdf]

S3 Table: Estimated ATE of Overall Remittance on Food Security Indicators using IPW and corresponding 95% Confidence Interval

Indicator	Estimated ATE	Std. Error	95% CI	Scale
HDDS	0.290	0.026	(0.240, 0.340)	Difference
Calorie Intake	128.620	15.680	(97.889, 159.349)	Difference
Food Expenditure (%)	-1.323	0.241	(-1.796, -0.849)	Difference
FIES Score	0.787	0.009	(0.769, 0.805)	Relative Risk

Notes: HDDS = Household Dietary Diversity Score (0-12); Calorie Intake = Per capita daily calorie intake (kcal/day); Food Expenditure (%) = Food expenditure as a percentage of total expenditure (0-100); FIES = Food Insecurity Experience Scale (0-8).
